# Effectiveness of the Integrated Dengue Education and Learning (iDEAL) module in improving the knowledge, attitude, practice, environmental cleanliness index, and dengue index among schoolchildren: A randomised controlled trial protocol

**DOI:** 10.1371/journal.pone.0302736

**Published:** 2024-04-30

**Authors:** Rahmat Dapari, Kalaivani Muniandy, Ahmad Zaid Fattah Azman, Suhaili Abu Bakar, Mohd Nasir Mohd Desa, Lim Chee Hwa, Sukhvinder Singh Sandhu, Nooreen Farzana Mustapha, Norazman Mohd Rosli, Mohd ‘Ammar Ihsan Ahmad Zamzuri, Mohd Rohaizat Hassan, Nazri Che Dom, Syed Sharizman Syed Abdul Rahim, Balvinder Singh Gill, Nurulhusna Ab Hamid

**Affiliations:** 1 Faculty of Medicine and Health Sciences, Universiti Putra Malaysia, Serdang, Malaysia; 2 Selangor State Health Department, Ministry of Health Malaysia, Shah Alam, Selangor, Malaysia; 3 Kuala Lumpur and Putrajaya Health Department, Ministry of Health Malaysia, Kuala Lumpur, Malaysia; 4 Negeri Sembilan State Health Department, Seremban, Seremban, Negeri Sembilan, Malaysia; 5 Department of Community Health, Universiti Kebangsaan Malaysia, Cheras, Malaysia; 6 Faculty of Health Sciences, Universiti Teknologi MARA, Bandar Puncak Alam, Malaysia; 7 Faculty of Medicine and Health Sciences, Universiti Malaysia Sabah, Kota Kinabalu, Malaysia; 8 Institute for Medical Research, National Institutes of Health (NIH), Ministry of Health Malaysia, Shah Alam, Selangor, Malaysia; 9 Medical Entomology Unit, Institute for Medical Research, National Institutes of Health (NIH), Ministry of Health Malaysia, Setia Alam, Selangor, Malaysia; Pharmaceutical Services Division, MALAYSIA

## Abstract

**Background:**

Dengue is a mosquito-borne disease caused by four distinct, closely related dengue viruses (DENV). Global dengue incidence has markedly increased in the past decades. The World Health Organization reported that cases increased from 505,430 in 2000 to 5.2 million in 2019. Similarly, the total dengue cases in Malaysia increased from 7,103 in 2000 to a peak of 130,101 in 2019. Knowledge, attitude, and practice (KAP) remain the most effective dengue prevention and control tools. Furthermore, school-based health education is key to enhancing knowledge and raising awareness of the seriousness of dengue among schoolchildren and transferring knowledge and practice from classrooms to homes. Thus, it is necessary to plan an integrated module for the primary prevention of dengue infection, specifically among schoolchildren.

**Aims:**

The present study intends to develop, implement, and evaluate the effectiveness of a theory-based integrated dengue education and learning (iDEAL) module in improving the KAP, environmental cleanliness index, and dengue index among schoolchildren in Selangor and Kuala Lumpur.

**Methods:**

This study is a single-blinded, cluster randomised controlled trial to be conducted from 1 September 2023 to 31 August 2025. The study will involve 20 primary and 20 secondary schools in Selangor and Kuala Lumpur. The 1600 participants will be randomly allocated to intervention and control groups based on selected clusters to avoid contamination. A cluster is a comparable school that fulfils the inclusion and exclusion criteria. The intervention group will receive the iDEAL module, while the control group will receive standard education. The iDEAL module will be developed following a systematic procedure and delivered in-person by trained researchers to the participants. The outcome will be measured using validated, self-administered questionnaires at baseline (T_0_), immediately (T_1_), one month (T_2_), and three months (T_3_) post-intervention to measure the intervention module effectiveness. The data will be analysed using IBM Statistical Package for Social Science (SPSS) version 28 and descriptive and inferential statistics. Within-group changes over time will be compared using one-way repeated measure analysis of variance for continuous and normally distributed variables. Within-group analysis of categorical data will use Cochran’s Q test. The main effect and interaction between and within the intervention and control groups at T_0_, T_1_, T_2_, and T_3_ will be tested using the generalised linear mixed model (GLMM). Hypothetically, the KAP, environmental cleanliness index, and dengue index among the intervention group will be significantly improved compared to the control group. The hypothesis will be tested using a significance level with a p-value of 0.05 and a confidence interval of 95%.

**Conclusions:**

The study protocol outlines developing and testing an iDEAL module for schoolchildren in Selangor and Kuala Lumpur, with no socio-demographic differences expected. The intervention aims to improve KAP, environmental cleanliness index, and dengue index, potentially reducing dengue risk. Results could inform public health policies, emphasizing school-based interventions’ importance in combating diseases like dengue.

## Introduction

Dengue is a disease caused by four distinct, closely related dengue viruses (DENV) and transmitted to humans through infected mosquito bites [1]. Dengue imposes a substantial burden on many tropical and sub-tropical regions [2]. Furthermore, the recent global dengue incidence has increased markedly. The World Health Organization (WHO) reported that cases increased from 505,430 in 2000 to 5.2 million in 2019 [1]. Moreover, the total dengue cases in Malaysia increased from 7,103 in 2000 to a peak of 130,101 in 2019 [3, 4]. As the previous peak was in 2019 and based on a cyclical pattern that peaks every four to five years, dengue is expected to peak in Malaysia in 2024 [3]. Selangor and Kuala Lumpur recorded the highest number and incidence of dengue cases and deaths in Malaysia.

The dengue clinical manifestations vary from mild illness to severe complications, where people who contract a secondary infection caused by a different DENV serotype have a higher risk of severe disease [5]. The initial dengue symptoms resemble those of other febrile illnesses and frequently result in misdiagnosis and incidence underestimation [6, 7].

The economic and resource burden on health services remains substantial in dengue-endemic settings [8]. Global estimates stated that there were 58.4 million symptomatic dengue virus infections in 2013, which included 13,586 fatal cases, and dengue illness reflected a total annual global cost of $8.9 billion [9]. Similarly, dengue infection imposes a significant economic burden in Malaysia. A 2013 study reported that the economic burden of dengue illness was $102.25 million annually and approximately $3.72 per capita [10]. The overall economic burden of dengue would have been even higher had the study included the costs associated with dengue prevention and control, dengue surveillance, and long-term dengue sequelae [10].

Accordingly, knowledge, attitude, and practice (KAP) remain the most effective tools against dengue infection [11–14]. School-based health education is key to enhancing knowledge, raising the awareness of the seriousness of dengue among schoolchildren, and transferring knowledge and practices from classrooms to homes [15]. Thus, it is necessary to plan an integrated module for the primary prevention of dengue infection among schoolchildren.

The authors intend to develop, implement, and evaluate the effectiveness of a theory-based integrated dengue education and learning (iDEAL) module in improving the KAP, environmental cleanliness index, and dengue index among schoolchildren in Selangor and Kuala Lumpur. The specific objectives are to develop the iDEAL module, determine and compare the baseline sociodemographic, socioeconomic, and dengue infection history between intervention and control groups, and evaluate the within- and between-group effects of the iDEAL module in improving the KAP, environmental index, and dengue index at baseline (T_0_), immediately (T_1_), one month (T_2_), and three months (T_3_) post-intervention.

## Methods

### Study design

This study is a two-arm, randomised, single-blinded, controlled parallel trial. Schoolchildren will be recruited and randomised to intervention and control groups based on selected clusters to avoid contamination. A cluster is defined as a comparable classroom based on a selected school that fulfils the inclusion and exclusion criteria. The intervention group will receive the iDEAL module, while the control group will receive standard education.

### Study duration

**Fig 1:**
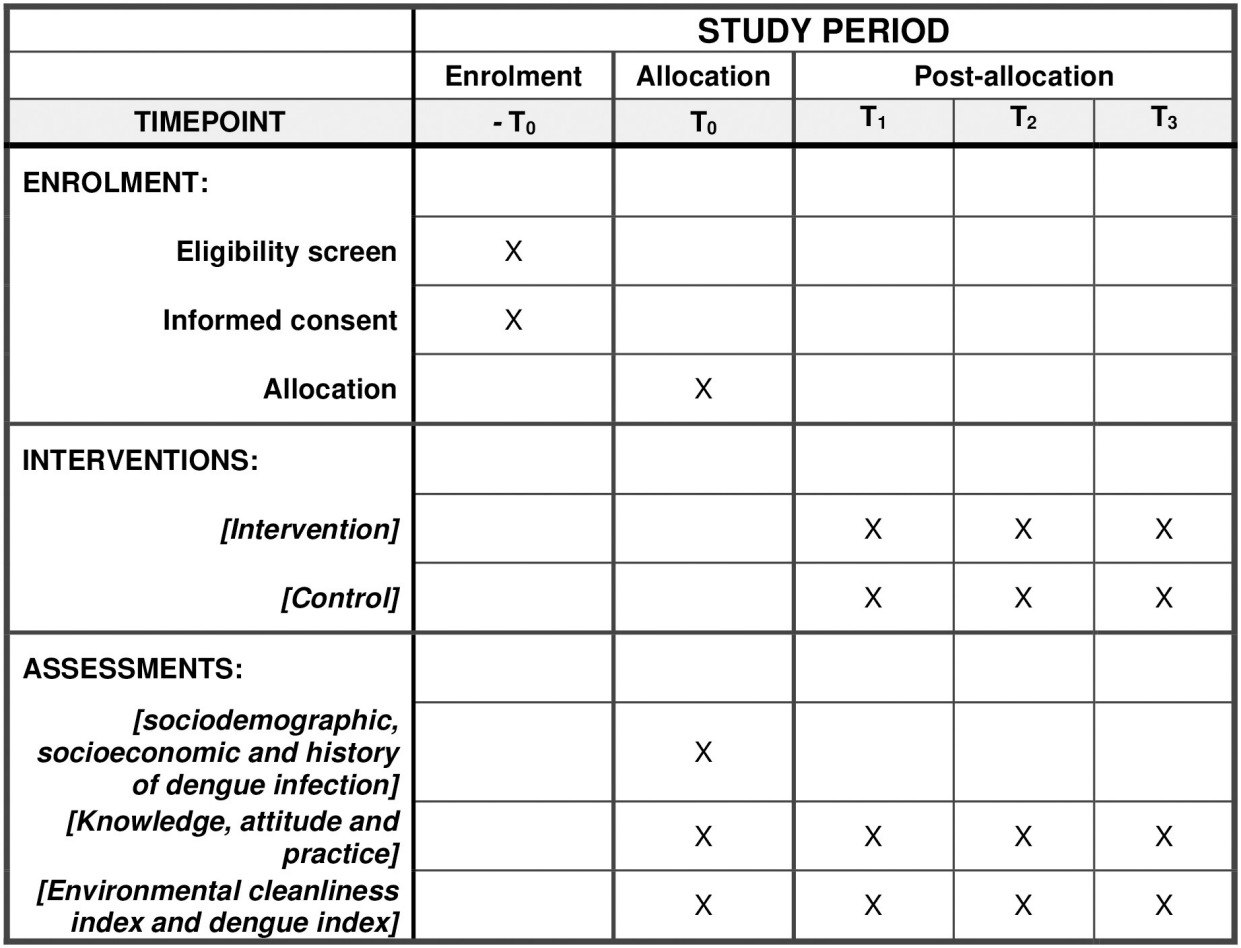
Enrolment, intervention, measurement, and assessment schedule.

The study will be conducted over 24 months, from 1st September 2023 to 31st August 2025. Data will be collected from 1st November 2023. The study will comprise research planning, iDEAL module development, implementation, and evaluation of the intervention effectiveness. Fig 1 depicts the enrolment, intervention, and measurement schedule and corresponding assessment times.

### Study location

The study will be conducted in Selangor and Kuala Lumpur, which are dengue hotspots that recorded the most dengue cases in Malaysia [16]. Selangor (latitude 2° 35’–3° 60’ N, longitude 100° 45’–102° 00’ E) has a land area of 7,945 km^2^ and is one of the most developed and highly populated states in Peninsular Malaysia [17, 18]. Kuala Lumpur (3° 8’ 28.3” N, 101° 41’ 11.5” E) is located on the west coast of Peninsular Malaysia with a land area of 243 km^2^ [17]. In 2020, the Selangor population was 6,994,423 with a population density of 880/km^2^. Children comprised 29.5% of the Selangor population. The Kuala Lumpur population numbered 8,622,000 (population density: 8157/km^2^), which rendered it the most densely populated and urbanised location in Malaysia [17]. Kuala Lumpur is the Malaysian capital and a federal territory in Selangor, which significantly influences its social and economic development. Furthermore, the ecological flows, patterns, and processes of Selangor and Kuala Lumpur are interlinked. Therefore, the analysis will include Kuala Lumpur.

### Study population

The Ministry of Education (MOE) Malaysia will supervise this study. The participants will be Standard 4 and Form 4 students from primary and secondary government schools, respectively, in Selangor and Kuala Lumpur. There are 661 primary schools and 279 secondary schools in Selangor, and 218 primary schools and 125 secondary schools in Kuala Lumpur. The schools include national, technical, science, religious, and boarding schools [19].

### Inclusion and exclusion criteria

The school inclusion criteria in this study are presented as follows: i) Selangor and Kuala Lumpur primary and secondary schools, ii) MOE-supervised government school, and iii) MOE permission. The exclusion criteria are presented as follows: i) school for special needs students, and ii) the distance between selected schools < 10 km.

The participants inclusion criteria are presented as follows: i) Malaysian citizen, ii) Standard 4 or Form 4 student, iii) parent or caregiver consent for student’s participation, and iv) school consent. The participants exclusion criteria are presented as follows: i) unable to read Bahasa Malaysia or English, ii) physically or mentally impaired and holding a disability card, and iii) temporary student (< 6 months at the involved school) during the study period.

### Sample size estimation

The sample size was calculated based on the desired primary outcome, namely knowledge of dengue prevention and control. The sample size was calculated using the formula for testing the difference in proportions between two groups [20]. Adjustment for a 70% attrition rate established that the sample size required for this study is 800 pairs of intervention and control groups, which amount to 1600 participants.

### Sampling method and participant recruitment

This study will use multistage sampling. The first sampling stage will use proportionate simple random sampling to select 40 schools based on the Selangor and Kuala Lumpur districts. There are nine districts in Selangor and 11 districts in Kuala Lumpur. Thus, all 20 districts in Kuala Lumpur and Selangor will be included for the proportionate simple random sampling to select 40 schools. The school selection sampling frame will be the list of Selangor and Kuala Lumpur schools according to primary and secondary schools from 20 districts. For this exercise, the sampling frame A will consist of primary school while sampling frame B will consist secondary school. This sampling frame to ensure the required 20 schools for Standard 4 (primary school) and 20 school from Form 4 (secondary school) can be obtained. In the second sampling stage, one classroom from each school selected from the first sampling stage will be chosen using simple random sampling. These samplings will yield 20 Standard 4 classrooms and 20 Form 4 classrooms. Subsequently, the sampling frame will be the Standard 4 and Form 4 classroom list from each selected school. All students in the selected classrooms will be invited to participate in the study.

### Randomisation

School cluster randomisation will be used to ensure administrative efficacy, reduce risk of experimental contamination, and enhance participant compliance. The Standard 4 and Form 4 classrooms will be randomly divided into intervention and control groups. The randomisation unit will be 1:1 in the control or intervention group. Contamination due to geographical issues will be minimised by setting the distance between each school to a minimum of 10-km radius from each other. A school will be randomly reselected from the sample frame to replace any school that does not fulfil this criterion.

### Sequence generation

Upon recruiting the schools and participants that fulfil the eligibility criteria, schools will be allocated to intervention or control groups using permuted block randomisation. The approach will ensure that closely balanced numbers of schools will be allocated to each study arm. Variable block sizes of 4, 6, and 8 will be used to blind the researcher from predicting the school allocation process.

### Allocation concealment

A randomisation list will be generated using “Create a block randomisation list” (Sealed Envelope Ltd., 2019). The main investigator will conduct the sequence generation and allocation concealment. Trained researchers will implement the study, which includes participant enrolment, envelope opening, and intervention administration in accordance with the CONSORT statement [21]. The list will include unique software-generated allocation codes consisting of two letters, followed by ≥ 1 digits (BW2, AM4, AB1, etc.). The codes will represent the intervention or control group. Only the sequence generator will be aware of the meaning of the codes to ensure allocation concealment. Subsequently, participants will be recruited using sequentially numbered, opaque, and sealed envelopes. The codes will be printed, cut out, and sealed in opaque envelopes. Subsequently, the envelopes containing the randomisation codes will be drawn by the liaison officer at each participating school. The randomisation codes will be released after schools are recruited for the study, which would occur following the completion of the baseline measurement. Thus, the parties involved during the randomisation will be blinded to the group to which the schools will be allocated before the codes are revealed.

### Blinding

This study will use single-blinding, where the participants will not be aware of their group status. Single-blinding aims to minimise performance bias (the Hawthorne effect), where the participant might alter their responses or behaviour if they are aware of their allocated group. It is not be possible to blind the field researcher, who will deliver the intervention module to the participants. Participant single-blinding is possible in this study, as the participants will not know whether they are receiving the intervention module or standard care. The intervention module is defined as the iDEAL module in addition to standard care, while standard care is defined as the current programme conducted by schoolteachers, district health offices, or local authorities. The intervention and control groups will receive standard care, as it is part of the dengue prevention and control programme established by the local health authorities.

## Research phase and tools

**Fig 2:**
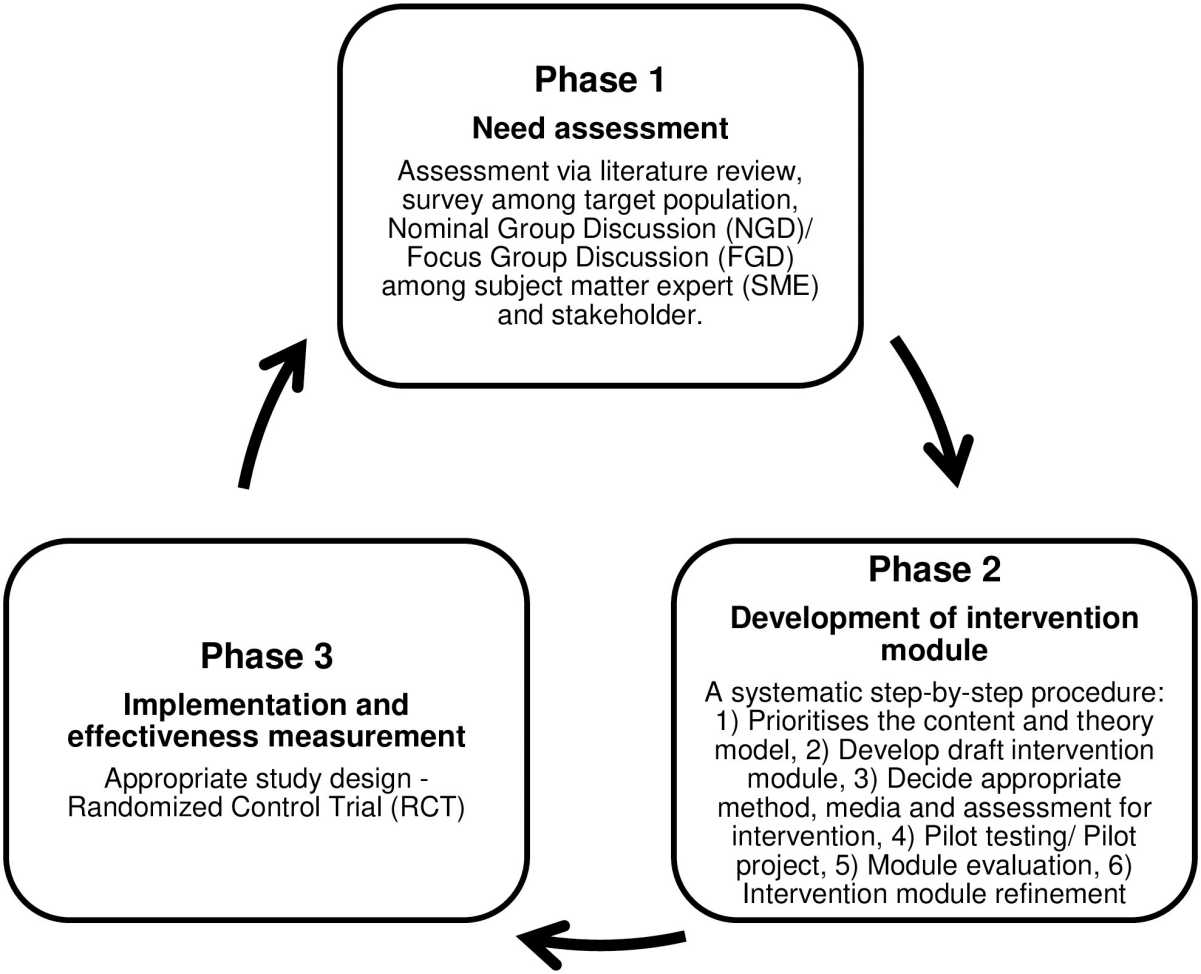
The three phases of intervention module development (3P-IMD).

This research protocol details the development, implementation, and effectiveness measurement of the iDEAL module in improving the KAP, environmental cleanliness index, and dengue index among schoolchildren in Selangor and Kuala Lumpur. This study consists of three phases (Fig 2).

### Phase I: Needs assessment

The needs assessment of the intervention will be conducted through the combination approach of community diagnosis. The KAP regarding dengue prevention and control will be assessed using a literature review, survey, nominal group discussion (NGD), and focus group discussion (FGD) among subject matter experts (SME) and stakeholders. All information will be used for module development (Phase II) to design the delivery, content, and assessment method and media.

### Phase II: Intervention module development

The iDEAL module will be systematically designed and developed. Table 1 outlines the six steps of the intervention module development.

**Table 1:** The intervention module development procedure.

Step	Objective	Activities
1.	Prioritises the content and theory model	NGD and FGD among SME (Fuzzy Delphi method will be used to rank and prioritise)
2.	Develop draft intervention module	Development group Review committee
3.	Determine appropriate intervention method, media, and assessment	Development group Review committee SME (Fuzzy Delphi method will be used to rank and prioritise)
4.	Pilot testing or pilot project	Small-scale preliminary study conducted to evaluate: feasibility duration cost adverse events
5.	Module evaluation	Evaluation: Context, input, process, and product (CIPP)
6.	Intervention module refinement	Incorporate feedback from pilot training and module evaluation to finalise the intervention module

### Phase III: Implementation and effectiveness measurement

The final module will be implemented at the selected schools. A trained researcher will conduct the intervention to ensure standardisation, which will be optimised using a pre-recorded video, briefing, and serial supervision by the principal investigator throughout the intervention period. The intervention module effectiveness will be measured using validated self-administered questionnaires at T_0_, T_1_, T_2_, and T_3_.

## Questionnaire

The primary study outcome will be the KAP score, which is measured with validated self-administered questionnaires at T_0_, T_1_, T_2_, and T_3_ post-intervention. The questionnaire will be printed in English and Malay. Furthermore, the questionnaire is a new instrument that will be developed and validated based on various combinations of questionnaires on similar topics. The questionnaire will be divided into the following parts:

Part 1: Participants information, which consists of questions on socio-demographics, socio-economics, and dengue infection history;
Part 2: Dengue prevention and control knowledge;
Part 3: Dengue prevention and control attitude;
Part 4: Dengue prevention and control practices.

The secondary study outcome will be the environmental cleanliness index and *Aedes* index, which trained researchers will measure using an observational checklist tool at T_0_, T_1_, T_2_, and T_3_ post-intervention.

## Validity and reliability

The content validity of the questionnaire will be assessed by SME, which will include a public health medicine specialist, family medicine physicians, health educators, health promoters, and school teachers from various agencies. The questionnaire acceptability will be assessed by calculating the content validation ratio (CVR). Furthermore, the SME comments and feedback will be considered for further correction of the questionnaire. Additionally, the assigned experts will assess the questionnaire relevance and clarity by calculating the content validity index (CVI). Subsequently, the Item CVI (I-CVI) and the scale average CVI (S-CVI/Ave) will be calculated. The questionnaire will be modified according to the experts’ agreement level based on the calculated CVI. Face validation among the schoolchildren will be conducted to ensure their accurate understanding of the meaning underlying each item and the feasibility of the data collection period.

The instrument reliability will be measured using the internal consistency method calculated in IBM Statistical Package for Social Science (SPSS) version 28. The sample size for the instrument reliability study will be calculated using a web-based sample size calculator (https://wnarifin.github.io/ssc_web.html). Subsequently, participants will be recruited to achieve the sample size. All participants recruited for the instrument reliability test will be excluded from the main study. The internal consistency of the scale will be calculated using Cronbach alpha coefficient (a Likert scale) or Kuder-Richardson coefficient (items with a dichotomous response).

## Data collection

**Fig 3:**
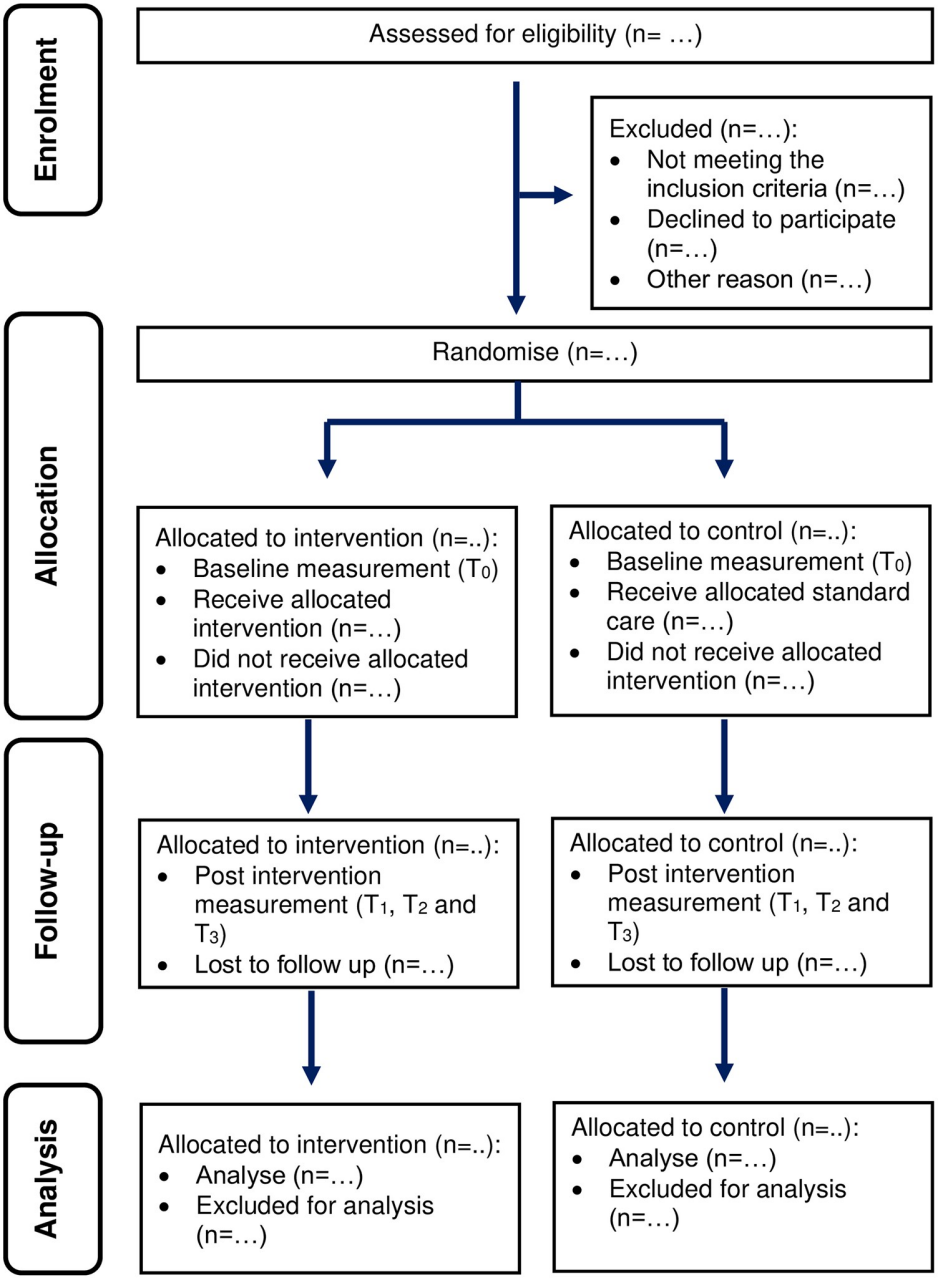
Flow diagram of study based on the CONSORT statement.

Upon initiating the study, the liaison officer will obtain consent from the students who fulfil the eligibility criteria. Subsequently, T_0_ data will be obtained from the students who consent to participate in the study. The participants allocated to the intervention group will be given appointments within one month for intervention package delivery according to class. The total duration of the intervention sessions will be approximately 30 minutes in three meetings. After completing the intervention, the participants will be required to answer the post-intervention assessment measuring the study outcome at T_1_, T_2_, and T_3_. The control group will undergo the same assessment as the intervention group at T_0_, T_1_, T_2_, and T_3_. Fig 3 depicts the study flow diagram based on the CONSORT 2010 statement [20].

## Data analysis

The data analysis will involve the intention-to-treat analysis strategy. The approach analyses the results in a randomised study, where all randomised participants are included in the statistical analysis and analysed according to the group they were originally assigned, regardless of the treatment received.

The collected data will be analysed using IBM SPSS version 28 and descriptive and inferential statistics. Data exploration will be performed to identify data entry errors, missing data, and outliers. Subsequently, the numerical data will be identified as normally or non-normally distributed via statistical and graphical methods. The statistical method will involve calculating the skewness or standard error (SE) of skewness and the Kolmogorov-Smirnov parameter, while the graphical method will involve plotting and analysing histograms and stem and leaf box plots. Normally distributed data will be described using mean and standard deviation, while non-normally distributed data will be described using median and the first and third quartiles (Q1, Q3). Categorical data will be presented as frequency and percentage.

The hypothesis will be tested using a significance level with a p-value of 0.05 and a confidence interval of 95%. Results that yield a p-value < 0.05 will be considered statistically significant. For between-group comparisons, the mean difference of continuous and normally distributed data between the intervention and control groups will be compared using the independent sample *t*-test. For continuous but non-normally distributed data, the intervention and control groups will be compared using the Mann-Whitney U test. The frequency difference of categorical data between the intervention and control groups will be compared using the chi-square test. Fisher’s exact test will be used for data from 2 × 2 tables, which contain >20% cells with an expected count < 5.

Within-group changes over time will be compared using one-way repeated measure analysis of variance (ANOVA) for continuous and normally distributed variables. Cochran’s Q test will be used for within-group analysis of categorical data. The main effect and interaction between and within the intervention and control groups at T_0_, T_1_, T_2_, and T_3_ will be tested using the generalised linear mixed model (GLMM).

## Ethics approval, trial registration, and consent to participate

The Universiti Putra Malaysia Ethics Committee for Research Involving Human Subjects approved this study (JKEUPM-2023-347, dated 19 June 2023). This study has also been approved for National Medical Research Registration of Malaysia (NMRR) registration on 25 September 2023 (NMRR ID-23-02823-PKM). Additionally, the Thai Clinical Trial Registry (TCTR) approved clinical trial registry registration (reference number: TCTR20230426009, dated 26 April 2023). This study protocol has been registered with the ClinicalTrials.gov committee (ClinicalTrials.gov Identifier: NCT05863026, dated 8 May 2023). Written approval from the MOE Malaysia regarding each selected school will be obtained before data collection. The relevant parties will be informed of all protocol changes. All participants will be informed that their enrolment will not affect their relationship with their teachers or school. The researcher will obtain written consent from each participant’s parent or caregiver. The participants will be issued a consent form upon agreeing to participate in the study before answering the baseline questionnaire. The participants will be allowed to withdraw at any point during the study.

## Consent for publication and dissemination

The participants’ consent for publication will be obtained from the same participation consent form. Notably, the results will be reported and presented in international peer-reviewed journals, conferences, and other platforms. No personnel or school information will be disclosed in the dissertation writing and other published manuscripts.

## Supervisory committee

The supervisory committee will involve the researchers and stakeholders. The committee will monitor the study from its initiation to the end, monitor the data, and advise on the research methods and appropriateness regarding the study aims. Monthly meetings are planned, with supervisory committee members requested for further input, including trial conduct auditing, reviewing interim analysis results, and halting the guideline if needed.

## Results or trial status

The intervention module development SME and potential schools have been identified. The feedback and comments of the schools on the intervention module development will then be compiled. Serial NGD and FGD with the respective experts will be conducted following intervention module development Phase II.

## Data sharing

The research will be disseminated and knowledge translated by networking among stakeholders at the national level to broaden the health policy on using the iDEAL module in the school syllabus nationwide. Second, the researchers will engage with school teachers, parent–teacher associations (PIBG), and other co-curriculum teachers regarding iDEAL module usage. Subsequently, train-the-trainer (TTT) programmes will be conducted among the aforementioned parties to expand iDEAL module implementation nationwide. Third, the findings will be disseminated through the traditional academic routes of conferences and peer-reviewed publications.

## Strengths and limitations

This study will involve a systematic intervention module development that includes a literature review, need assessment for the relevant target population, and SME. This approach influences the intended outcome and ensures an effective end product. The risk of contamination will be minimised via cluster randomisation. Ensuring that the selected schools are 10 km away from each other would further minimise the contamination risk. Thus, the intervention module effect on the intended outcome can be precisely measured.

Although the module development, implementation, and effectiveness assessment measures in this study are robust, the intended KAP outcome measurements will be self-reported. A self-administered questionnaire remains an appropriate measurement in a community intervention study focusing on the individual influence on KAP. Additionally, the researcher will measure the environmental cleanliness index and dengue index, which will provide reliable and valid measurements.

One possible challenge in this study is limited enrolment. In this instance, the researchers will adjust the recruitment methods, extend the enrolment periods, and cooperate with other research institutions. Education, regular contact, reminders, support services, and incentives increase participant adherence. Pre-defined assessment, reporting, and intervention protocols will be established in the case of concurrent events. The effects of such events on the study will be monitored, documented, and evaluated.

The intervention and control groups might experience prevention and control activities by health authorities whenever the school experiences a dengue outbreak. Considering that these activities are part of the standard care that both groups could receive during the study period, this condition will be controlled during analysis and interpretation. Information on these activities will be collected from both groups. The research outcomes based on this condition will be carefully interpreted.

## Discussion and conclusion

This study protocol outlines the comprehensive process of developing and assessing the efficacy of an integrated dengue education and learning (iDEAL) module tailored for schoolchildren in Selangor and Kuala Lumpur. The participants’ socio-demographic characteristics are not expected to differ between the groups. Hypothetically, the intervention group will have significantly improved KAP, environmental cleanliness index, and dengue index compared to the control group. In the GLMM analysis, the iDEAL module could effectively improve the KAP, environmental cleanliness index, and dengue index among schoolchildren in Selangor and Kuala Lumpur. By empowering these young individuals with vital knowledge and fostering proactive practices, the study anticipates a tangible reduction in dengue infection risk within the targeted regions. Furthermore, the findings hold potential to influence broader public health policies, underscoring the significance of school-based interventions in combating mosquito-borne diseases like dengue fever.

## Supporting information

S1 Checklist SPIRIT checklist study protocol dengue module IDEM. (DOC)

S1 Table All items from the world health organization trial registration data set. (DOCX)
