# Supplementary material for: Effectiveness of the Integrated Dengue Education and Learning (iDEAL) module in improving the knowledge, attitude, practice, environmental cleanliness index, and dengue index among schoolchildren: A randomised controlled trial protocol
Source: PLoS One. 2024 Apr 30;19(4):e0302736. doi: 10.1371/journal.pone.0302736 (PMC11060578; doi:10.1371/journal.pone.0302736)
Supplement: S1 Table — (DOCX) [file pone.0302736.s002.docx]

Table: All items from the World Health Organization Trial Registration Data Set

|  | Primary registry and trial identifying number | Thai Clinical Trial Registry (TCTR) TCTR20230426009 |
| --- | --- | --- |
|  | Date of registration in primary registry | 26 April, 2023 |
|  | Secondary identifying numbers | ClinicalTrials.gov committee on 8 May 2023 with the ClinicalTrials.gov Identifier: NCT05863026 |
|  | Source(s) of monetary or material support | Takeda Malaysia Sdn. Bhd. |
|  | Primary sponsor | Universiti Putra Malaysia |
|  | Secondary sponsor(s) | Takeda Malaysia Sdn. Bhd. |
|  | Contact for public queries | Rahmat Dapari, MD, MPH, DrPH [+60192322815] [drrahmat@upm.edu.my] |
|  | Contact for scientific queries | Rahmat Dapari, MD, MPH, DrPH [+60192322815] [drrahmat@upm.edu.my] |
|  | Public title | Effectiveness of Theory Based Integrated Dengue Education and Learning Module (iDEAL) in Improving the Knowledge, Attitude, Practice, Environmental Cleanliness Index and Dengue Index Among School Children in Selangor and Kuala Lumpur: Study Protocol for A Randomised Controlled Trial |
|  | Scientific title | Effectiveness of Theory Based Integrated Dengue Education and Learning Module (iDEAL) in Improving the Knowledge, Attitude, Practice, Environmental Cleanliness Index and Dengue Index Among School Children in Selangor and Kuala Lumpur |
|  | Countries of recruitment | Malaysia |
|  | Health condition(s) or problem(s) studied | Knowledge, attitude, practice, environmental cleanliness index, and aedes index |
|  | Intervention | The intervention group: the intervention group will receive Theory Based Integrated Dengue Education and Learning Module (iDEAL)  The control group: active control will receive standard care from respective agency |
|  | Key inclusion and exclusion criteria | The inclusion criteria for the respondents in this study are:   1. Malaysian citizen, 2. standard 4 (age 10 y.o) or form 4 (age 16 y.o) from primary and secondary school respectively, 3. consented to participate in the study by parents or caregivers, 4. consented by the school.   The exclusion criteria are:   1. not able to read Bahasa Malaysia or English, 2. physically or mentally impaired with disability card holder, 3. temporary student as defined as less than 6 months during the study period. |
|  | Study type | Interventional Allocation: randomised Intervention model: parallel assignment Masking: single blind (subject) Primary purpose: prevention Phase 0 |
|  | Date of first enrolment | 1^st^ September 2023 |
|  | Target sample size | 1600 |
|  | Recruitment status | Pending: participants are not yet being recruited or enrolled at any site |
|  | Primary outcome(s) | Knowledge, attitude, practice on dengue prevention and control  Measure using validated, self-administered questionnaires at four time points: baseline (T_0_), Immediately (T_1_), one month (T_2_), and three months (T_3_) post-intervention |
|  | Key secondary outcomes | Environmental cleanliness index, and aedes index  Measure using observational checklist tool by trained researchers at four time points: baseline (T_0_), Immediately (T_1_), one month (T_2_), and three months (T_3_) post-intervention |
|  | **Ethics Review** | This study has been approved by the Ethics Committee for Research Involving Human Subjects of Universiti Putra Malaysia (JKEUPM-2023-347), dated 19 June 2023 |
|  | **Completion date** | Expected to be completed by 31^st^ May 2025 |
|  | **Summary Results** | Expected to be released by 31^st^ May 2025 |
|  | **IPD sharing statement** | The researcher committee has no plan to share IPD. However, the results will be reported and presented in international peer-reviewed journals, conferences and other platforms. No personnel or school information will be disclosed in the dissertation writing and other published manuscript. |
